# An approach to cellular tropism of SARS-CoV-2 through protein–protein interaction and enrichment analysis

**DOI:** 10.1038/s41598-022-13625-z

**Published:** 2022-06-07

**Authors:** Daniel Ortega-Bernal, Selene Zarate, Maria de los Ángeles Martinez-Cárdenas, Rafael Bojalil

**Affiliations:** 1 Department of Health Care, Universidad Autónoma Metropolitana, Unidad Xochimilco, 04960 Mexico City, Mexico; 2 Posgrado en Ciencias Genómicas, Universidad Autónoma de La Ciudad de México, Ciudad de México, Mexico City, 03100 México

**Keywords:** Computational biology and bioinformatics, Microbiology, Molecular biology, Molecular medicine

## Abstract

COVID-19, caused by SARS-CoV-2, is a primarily pulmonary disease that can affect several organs, directly or indirectly. To date, there are many questions about the different pathological mechanisms. Here, we generate an approach to identify the cellular-level tropism of SARS-CoV-2 using human proteomics, virus-host interactions, and enrichment analysis. Through a network-based approach, the molecular context was visualized and analyzed. This procedure was also performed for SARS-CoV-1. We obtained proteomes and interactomes from 145 different cells corresponding to 57 different tissues. We discarded the cells without the proteins known for interacting with the virus, such as ACE2 or TMPRSS2. Of the remaining cells, a gradient of susceptibility to infection was observed. In addition, we identified proteins associated with the coagulation cascade that can be directly or indirectly affected by viral proteins. As a whole we identified 55 cells that could be potentially controlled by the virus, with different susceptibilities, mainly being pneumocytes, heart, kidney, liver, or small intestine cells. These results help to explain the molecular context and provide elements for possible treatments in the current situation. This strategy may be useful for other viruses, especially those with limited reported PPI, such as a new virus.

## Introduction

Understanding the molecular interactions of SARS-CoV-2 with its host (cell) is necessary to identify the various impacts of the infection at the organismal level. Sequestration of a host protein by a SARS-CoV-2 protein, either to inhibit or to promote its function, can modify a given molecular process. Numerous protein–protein interactions (PPIs) have been reported by Gordon DE et al.^1^, Li et al.^2^, and Stukalov et al.^3^. They represent an overview of the PPI networks and molecular processes under the context of the cell line from which those authors obtained results (HEK293T, embryonic kidney and A549 human lung (carcinoma)), as well as some extrapolations. Using data from these investigators, simulated data, associations with interactomes of other viruses or experimental data, several groups have generated general molecular potential interactions between viral proteins and human proteins. Examples are Sarbecovirus-human protein–protein interaction network^4^; network-based model for defining the molecular aspects of pathogenic phenotypes in HCoV infections^5^; overview of coronavirus-host interactions^6^; cellular infection profile^7^; uncover structural and functional modules^8^ or an organotropism beyond the respiratory tract^9^.

**Figure 1:**
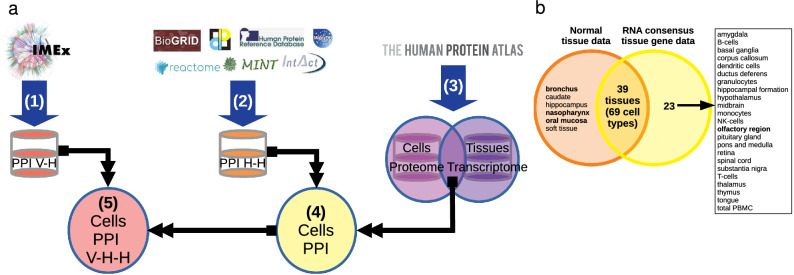
Research strategy. 1: (**a**) Obtaining the PPI of the SARS-CoV-2—Human (V-H) from IMEx; 2: Integration of human-specific PPIs from 7 primary databases (H–H); 3: Obtaining proteomes and transcriptomes, selecting those results that are reported at both levels; 4: Generation of human PPI (H–H) for each cell; 5: Obtaining the PPI of the viral infection (V-H–H) of each cell. Images were taken from https://www.imexconsortium.org/, https://dip.doe-mbi.ucla.edu/dip/Main.cgi, https://www.ebi.ac.uk/intact/, https://mint.bio.uniroma2.it/, https://thebiogrid.org/, https://www.hprd.org/, http://matrixdb.univ-lyon1.fr/, https://reactome.org/ and . (**b**) Tissues and cells that shared a set of the seven cell entry proteins/genes. For six cell types there are no reports at the tissue level (transcriptome) and for 23 tissues no cells are reported (proteome).

Just because a virus can enter a cell does not mean that there is an enabling environment for its replication; a coordinated cellular sequestration is necessary. We analyzed how 29 SARS-CoV-2 proteins interact with human proteins under their cellular context. We identified possible alterations of protein–protein interactions (PPI) by the infection within each cell, by searching for cells that have input mechanisms, already reported^10–18^ and for proteomes and interactomes within each cell associated with viral proteins. This resulted in identifying a group of cells with a certain degree of susceptibility to be controlled by SARS-CoV-2 (see Fig. 1). At the same time, we compared our findings related to SARS-CoV-2 with those we investigated for SARS-CoV-1.

## Results

### Input tropism

**Figure 2:**
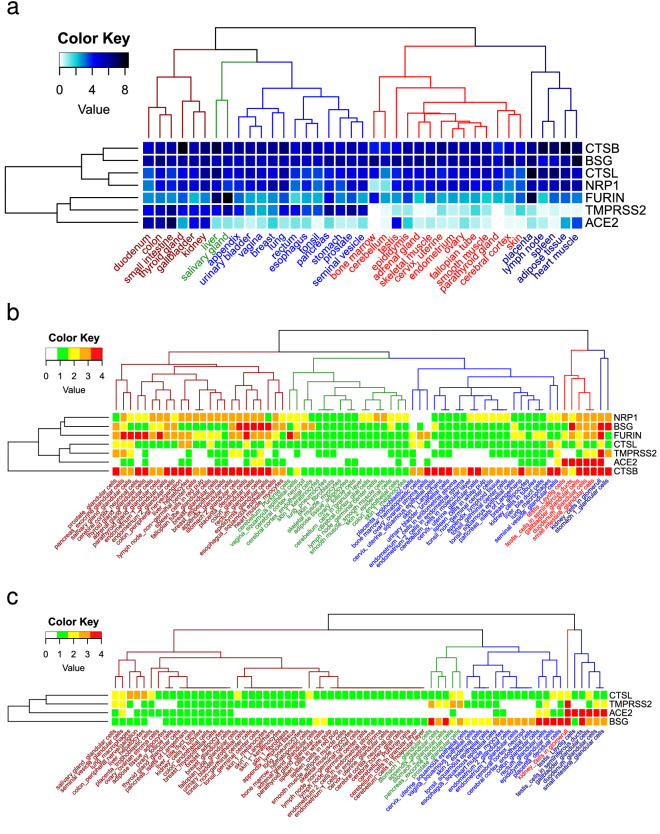
Cell and tissue heatmap based on potential input genes/proteins. (**a**) SARS-CoV-2, 39 tissues and seven messengers. Consistent with what is reported in the figure above, ACE2 and TMPRSS2 have the lowest expression, followed by FURIN. Of the 39 tissues, bone marrow, cerebellum and parathyroid gland have the lowest expression of ACE2 and TMPRSS2. The white to blue colored scales represent the Expression value provided by ProteinAtlas. (**b**) SARS-CoV-2, 69 cell types and seven proteins. In the red and dark blue branches and later in the dark red branch, the cell types with the highest protein presence are concentrated. Green and blue branches group those cell types with lower presence, as well as those without ACE2 or TMPRSS2 (also in the dark red branch), mainly. (**c**) SARS-CoV-1, 69 cell types and 4 proteins. The dark red branch is where the cells with the lowest presence of protein or mRNA are grouped; in the central part, the 14 removed cells are observed. The color represents the Expression value, white (not reported at either the transcript or protein level), green (only present at the transcript level), yellow (low amount of protein reported), orange (intermediate amount of protein reported), and red (high amount of protein reported).

The first step to identify cell tropism was to search for cells with potential entry-related proteins. We consulted two databases to cross-check the proteome and transcriptome data. First, from the RNA Consensus Tissue Gene Data we looked for those with reported expression of genes involved in cell entry: protein S-binding proteins (ACE2, NRP1, or BSG) and proteases (TMPRSS2, CTSL, CTSB, or FURIN). We selected 57 (out 62) tissues because they met the criterion of expressing at least 3 of the 7 genes we were looking for. Twenty threes (out 69) tissues (such as the olfactory region) had no information on cell types (Fig. 1b). Second, from the Normal Tissue Data, we found 45 tissues with at least 3 of the seven proteins of interest. Six cell types including those at bronchus, nasopharynx and oral mucosa did not have a transcriptome report at the tissue level (see Fig. 1b). Both data sets shared 39 tissues, including 69 different cell types (see Figs. 1b and 2a). For SARS-CoV-1 we considered the presence of four proteins (ACE2, BSG, TMPRSS2 and CTSL) to identify relevant tissues.

Of note, in 16 out of 69 (23%) cell types from 10 of the 39 tissues identified in the analysis for SARS-CoV-2, we didn’t find reports of ACE2 or TMPRSS2 protein, nor of their mRNA: adrenal gland (glandular cells), bone marrow (hematopoietic cells), cerebellum (granular layer cells, molecular layer cells and Purkinje cells), cerebral cortex (endothelial cells, glial cells, neuronal cells and neuropil), endometrium (glandular cells 1 and 2), germinal and non-germinal center cells of lymph nodes, parathyroid gland (glandular cells), skeletal muscle (myocytes), smooth muscle (smooth muscle cells) and spleen (red pulp cells), see Fig. 2b. Notably, bone marrow do not express ACE2 or TMPRSS2, and several tissues have values below the threshold (NX < 1) of ACE2, such as lung (0.8). Nonetheless, no cell type was removed from the analysis, since they expressed at least three of the necessary proteins to enter the cell. In contrast, for SARS-CoV-1, we removed 14 cell types with neither ACE2 nor TMPRSS2 (nor in mRNA); BSG and CTSL only had expression data from mRNA (see Fig. 2c). These results show that ACE2 and TMPRSS2 are the least common in the 69 cell types of the seven input proteins.

### PPI tropism

To investigate differences in the PPI networks that may occur in infected cells, we compared the size of the interactomes inferred in each cell type. The cells showing the highest number of interactions with SARS-CoV-2 proteins were located in the kidney (tubule cells and glomerulus cells), small intestine (glandular cells), and lung (pneumocytes), with a maximum of 970 proteins out of the 1345 reported. The least connected cells were located in the bone marrow (hematopoietic cells), colon (peripheral nerve/ganglion), placenta (decidual cells), and ovary (follicular cells), see Supplementary Table S1. For SARS-CoV-1, the cells with the fewer interactions were the same as in SARS-CoV-2, and the cells with the most interactions were lung (pneumocytes) and glandular cells (thyroid gland, duodenum, salivary gland, small intestine, gall bladder, colon, and pancreas), with 114 proteins (out of 151) interacting with the 26 viral proteins (see Supplementary Table S2).

When clustering the cell types by their expression value, several proteins were highly expressed in a group of cells, see Supplementary Fig. S1. Comparing the unsupervised hierarchical clustering (Supplementary Fig. S2), we found that mainly these cell types were glandular. A similar pattern was observed for SARS-CoV-1 (Supplementary Fig. S1c).

**Figure 3:**
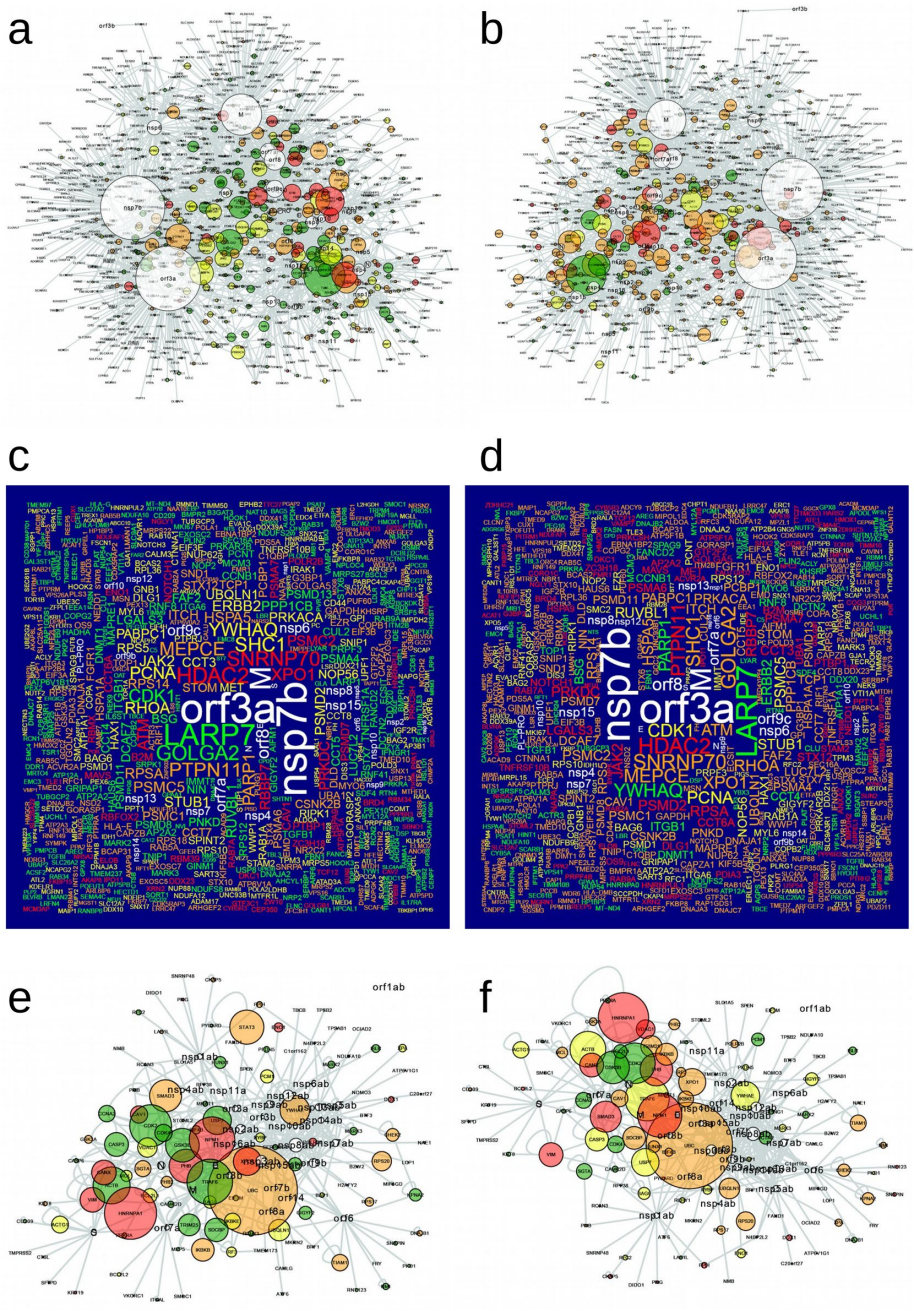
V-H networks and their respective wordclouds. (**a**,**c**) lung (pneumocytes); (**b**) and (**d**) lung (macrophages), in SARS-CoV-2; (**e**,**f**) networks in SARS-CoV-1, same order as the previous ones. The wordclouds show the main proteins present in (**a**,**b**). The size of the circles indicates the connectivity and the color to the Expression value, 1: green (only present at the transcript level), 2: yellow (low amount of protein reported), 3: orange (intermediate amount of protein reported), 4: red (high amount of protein reported) and in white the viral proteins.

This finding of a higher expression value in glandular cells than in pneumocytes, the main target cells, brought out the hypothesis that the susceptibility of a cell could depend not only on the expression profile but also on their connectivity to the viral proteins. Thus, we characterized the internal dynamics of the cells. We identified their connectivity according to their PPI, by assessing the Average Degree of each network (which ranges from 4.561 to 7.391). We found that the most connected cellular networks were indeed pneumocytes, and also glandular cells (endometrium 1), squamous epithelial cells (esophagus), non-germinal center cells (lymph node and tonsil tissues), and cells in the red pulp (spleen), see Supplementary Table S1. Networks visualized at the V-H PPI level highlighted emblematic cells, for example, the lung (pneumocytes and macrophages) as the most affected tissue (Fig. 3), and small intestine (glandular cells) as the tissue with the highest expression of ACE2 (Fig. S3a). Considering the expression in each cell and the total connectivity of each protein, our findings indicate that the human proteins with the most interconnections to viral proteins (V-H) were ATP1A1, ATP2A2, HACD3, XPO1 and ATP5F1B. The most linked proteins SARS-CoV-2 to human were M, nsp7b (orf7b) and orf3a, while the most connected within human cells (H–H) were LARP7 and GOLGA2. See in Supplementary Table S3 the connectivity of all proteins. Figure 3e,f and Fig S3c, show the networks for SARS-CoV-1, where UBC was the protein with the most connections, with a similar expression pattern as for SARS-CoV-2. See in Supplementary Table S4 the connectivity of each protein in general (not limited to a cell type) in SARS-CoV-1.

**Figure 4:**
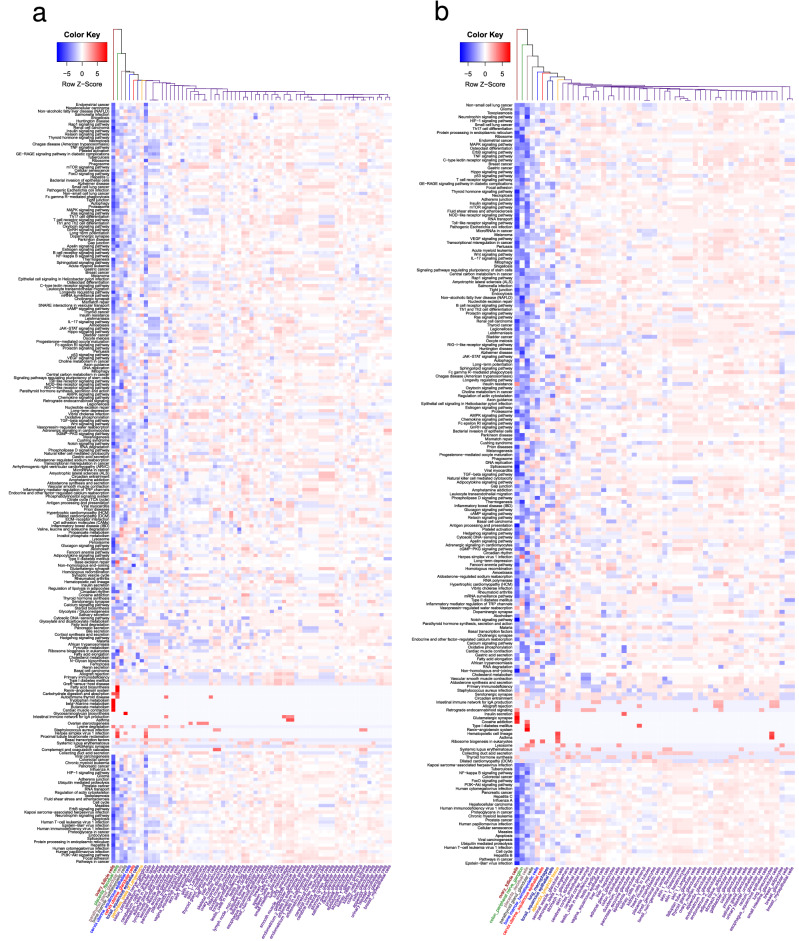
Enrichment of results, KEGG heatmap. (**a**) Signaling pathways in SARS-CoV-2; (**b**) signaling pathways in SARS-CoV-1. The blue color identifies a higher p-value and the red color a lower value (using a logarithmic transformation). A gradient of involvement of the signaling pathways, and therefore of the cells, is identified in both heatmaps from left to right (less altered to more altered, respectively).

We generated a gene set enrichment analysis (GSEA) for each proteome of each cell type to identify specific processes potentially altered by PPI. Unsupervised clustering (binary distance and simple linkage method) allowed the identification of a “gradient” of cells, where molecular processes were altered from lower to higher degree. Figure 4 shows the KEGG (Kyoto Encyclopedia of Genes and Genomes) signaling pathways in SARS-CoV-2 and SARS-CoV-1. In both heatmaps, we identified an effect of proteome size (Supplementary Table S1 y S2), thus 14 cell types with a small proteome were removed, reducing to 55 the initial 69 cell types. For SARS-CoV-1, lung macrophages and tonsil’s squamous epithelial cells were also identified as not significantly altering the molecular environment and were therefore also removed for subsequent analyses. Hence, for SARS-CoV-2, 55 cell types remained and for SARS-CoV-1, 45. Supplementary Figure S4 shows the behavior of the remaining SARS-CoV-2 cells in terms of molecular functions and biological processes. With these results, we identified that salivary gland (glandular cells), esophagus (squamous epithelial cells), testis (Leydig cells), lung (pneumocytes) and small intestine (glandular cells) behave as a group (identifiable in molecular functions, dark blue branch, where they share a group of unaltered functions). Supplementary Figure S5 shows the effect of this reduction on SARS-CoV-1.

**Figure 5:**
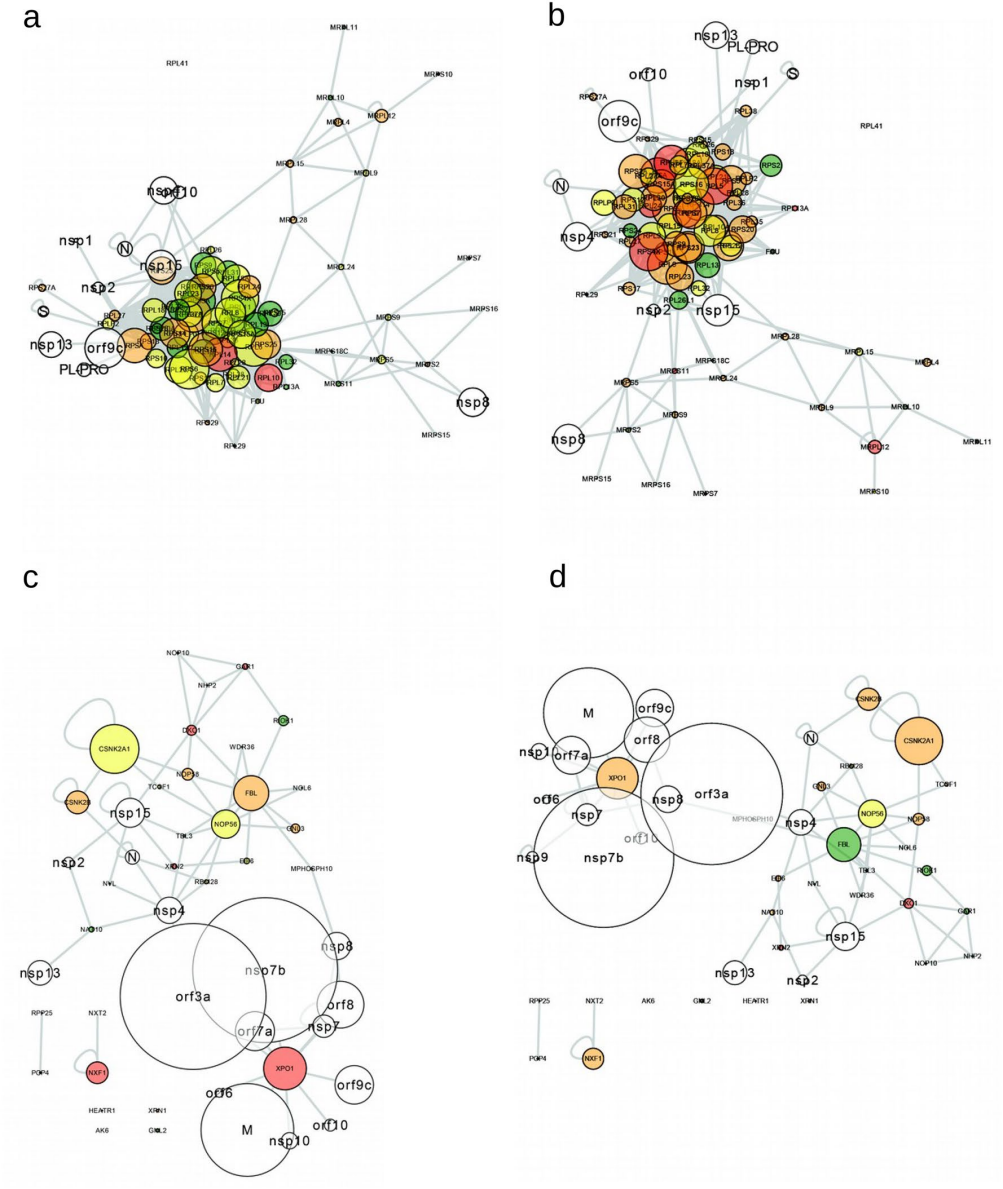
Specific networks. (**a**,**b**) ribosome pathway networks: (**c**,**d**) networks of the ribosome synthesis pathway. Lung (macrophages and pneumocytes), respectively. The color code and size nodes are the same as in Fig. 3. In the ribosome pathway there are 11 viral proteins (N, nsp4, PL-PRO, nsp2, nsp8, nsp13, orf9c, S, nsp1, orf10, and nsp15) that interact with proteins of this pathway. In Ribosome biogenesis in eukaryotes pathway there are 16 viral proteins (nsp7b, N, orf7a, nsp4, nsp2, nsp8, nsp13, nsp7, orf8, nsp10, orf9c, M, orf6, orf10, nsp15, and orf3a) that interact with proteins in this pathway; among them the 3 proteins with a high connectivity of SARS-CoV-2: M, nsp7b, and orf3a. These data are for the 2 cells shown. Similar results for kidney (cells in tubules) and small intestine (glandular cells) are present in Supplementary Fig. S7.

**Figure 6:**
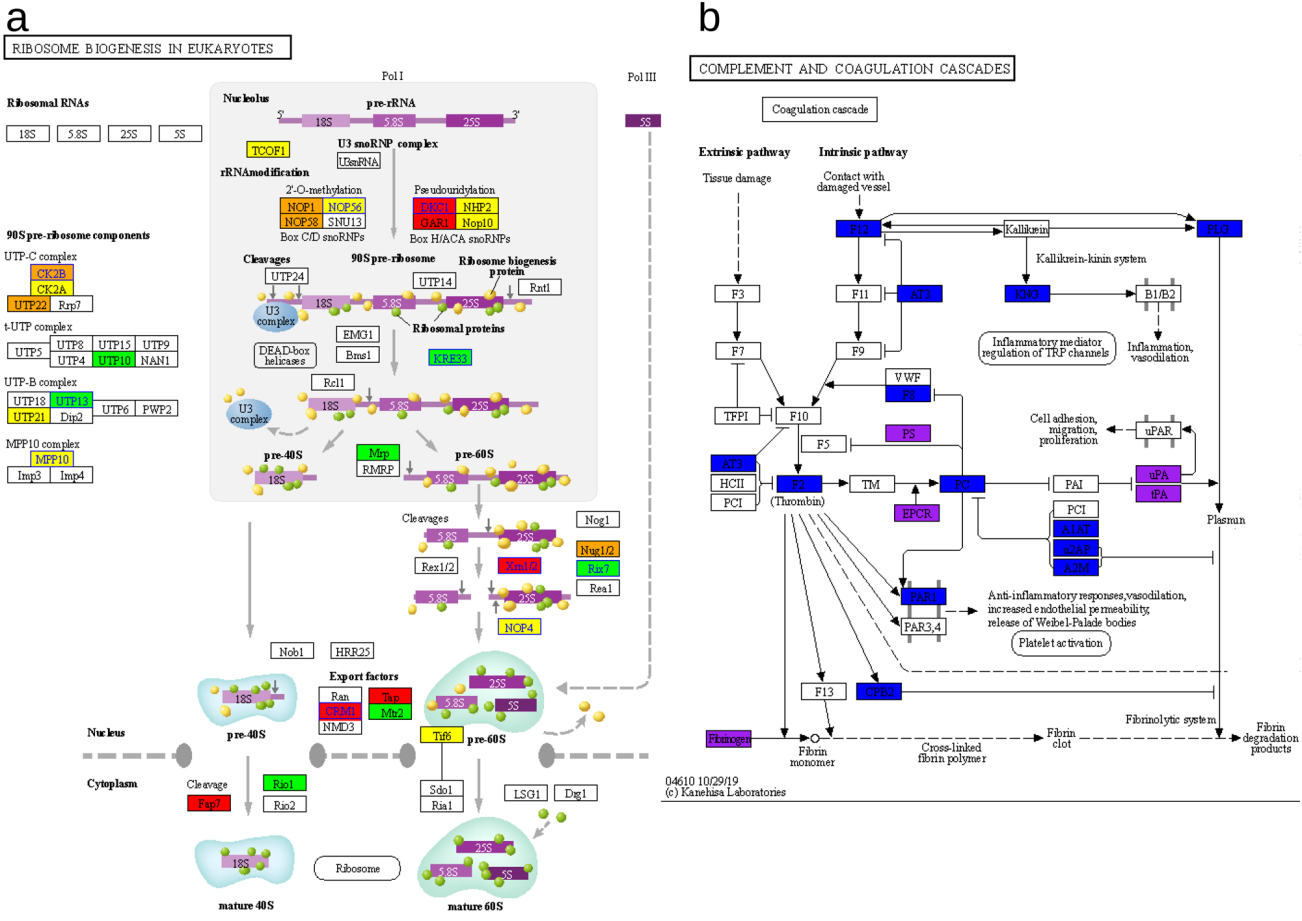
Signaling pathways KEGG^19^. (**a**) Signaling pathway for the synthesis of ribosomes in lung (pneumocytes), 10 proteins with direct interaction with name and line blue. 30 of 101 proteins are reported. The colors code associated to expression value was used. (**b**) Coagulation cascade and the 19 proteins (39 including those of the complement system) that can interact directly (purple), 6 proteins, or indirectly (blue), 13 proteins, with SARS-CoV-2 proteins in blood. Only the coagulation cascade is shown.

Supplementary Figure S6 shows the 50 most discriminating results, and the profiles of the gradients shown in the previous results can be identified. For SARS-CoV-2, one signaling pathway that appears relevant is "ribosome biogenesis in eukaryotes" (Supplementary Fig. S6b). Given its relevance, we generated subnetworks of ribosomes and their biogenesis. The ribosome network showed high protein expression value in the small intestine (glandular cells); moderately-high in the lung (macrophages); moderate in the kidney (tubule cells), and moderately-low in the lung (pneumocytes) (see Fig. 5a,b, and Supplementary Fig. S7a and b). We identified that pneumocytes and glandular cells of the small intestine behave similarly in some Expression Values, as is the case of XPO1, which extensively connects with SARS-CoV2 proteins (see Fig. 5c,d, and Supplementary Fig. S7c and d). We found that XPO1, CSNK2B, XRN2, DKC1, NOP56, NVL, RBM28, NAT10, TBL3, and MPHOSPH10 interact directly with virus proteins; and other 20 proteins do so indirectly (Fig. 6). These results suggest that SARS-CoV-2 hijacks and appears to promote ribosome synthesis, which would potentially enhance their replication capability.

In addition, we found that coagulation cascade proteins such as FGA, FGB, PROS1, PLAU, PLAT, PROCR, and CPB2 may also interact directly with virus proteins (Figs. 4a, 6b).

**Figure 7:**
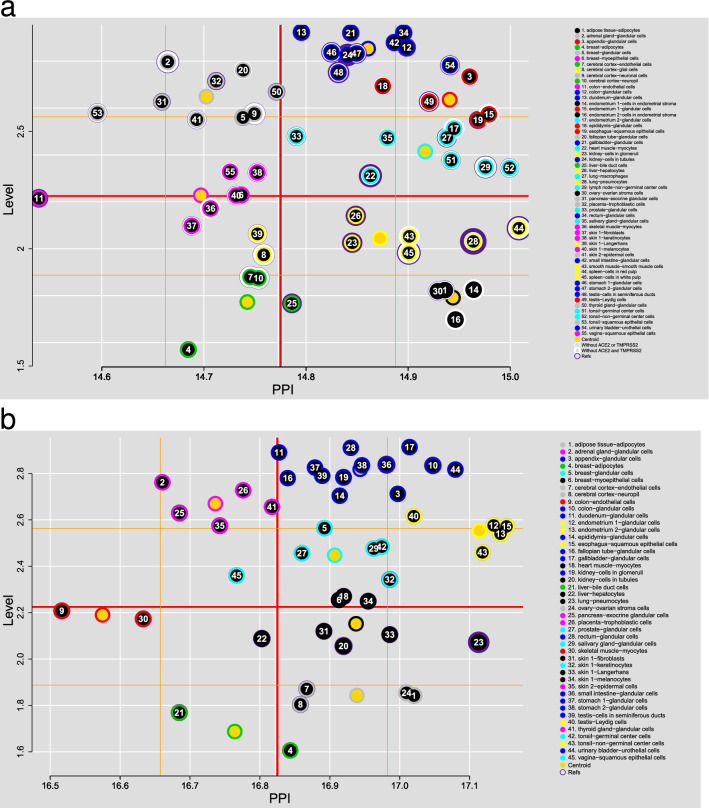
K-Means of the cells and their potential of infection. (**a**) SARS-CoV-2; at the top of quadrant 4, the cells most reported in the literature as infected in autopsies are identified (width purple ring), followed by quadrant 1. (**b**) SARS-CoV-1; pneumocytes are found in the same region as in (**a**) (upper side of quadrant 4, black group). Quadrant 1: high expression value (Level) and high PPI (upper right side); quadrant 2: high expression value and low PPI (upper left side); quadrant 3: low expression value and low PPI (bottom left); and quadrant 4: low expression value and high PPI (lower right side). As expected, glandular cells are mainly concentrated in the upper quadrants, gray, blue, red, and cyan cluster in (**a**) and lilac, blue, cyan, and yellow in (**b**). For Ref., see Supplementary Table S1, S2 and Supplementary Information.

Finally, from the centroids of 55 SARS-CoV-2 cell types (45 for SARS-CoV-1), we identified 8 clusters separated into 4 quadrants (Fig. 7). According to the distribution of both the Expression value and the PPI, we identified the importance of associating the potential interactions with the level of protein expression. Most of the cells of the most cited tissues in the literature, such as pneumocytes, kidney (tubule cells and glomerulus cells), testis (seminiferous duct cells), heart muscle (myocytes) and liver, are found in quadrants 1 and 4 (high PPI) (see Supplementary Tables S1 and S2).

## Discussion

In this work, we analyzed human PPI and proteome, GSEA information, and reported virus-host protein interactions to identify cells and tissues with the putative necessary environment to sustain infection by SARS-CoV-2. This analysis identified a series of tissues and cell types that could potentially be a target for viral infection. Several studies have been carried out to determine the breadth of SARS-CoV-2 cell tropism outside the respiratory tract (e.g., lungs and trachea). Still, many have evaluated the permissiveness of various cell lines, which is hard to interpret. Autopsy analyses have shown the presence of SARS-CoV-2 antigens, by immunodetection, in the trachea, small intestine, kidneys, pancreas, and vascular tissues of the brain and heart^20,21^. Our work identified possible targets for infection, in addition to cells of the respiratory tract, the small intestine and pancreas. Other studies have pointed to the potential infection of these tissues. Interestingly, Stillfried et al. using pathological specimens, have identified SARS-CoV-2 by RT-PCR in different tissues, including small intestine^22^.

It was initially difficult for us to identify a set of cells characteristically susceptible to SARS-CoV-2 infection. The amount of ACE2 in the tissues has not shown a correlation with infection or with TMPRSS2 in any of the reported organs^15,16,18^. Therefore, we decided to explore all 7 proteins already known to participate in viral entry, such as NRP1^10^, that could explain the tropism to different organs. This strategy did not show a better correlation with the organs known to be affected by SARS-CoV-2. Thus, the next step was to explore the intracellular molecular context, to determine the interactome and signaling pathways.

A high expression profile was supposed to be linked to a greater susceptibility to infection, but as the overexpression is mainly linked to glandular cells, which was already reported^23,24^, other factors should be considered. It is plausible that a higher concentration of cellular proteins increases the possibility of an interaction with viral proteins, since competition for protein interaction could play an important role. This possibility should be explored but to date there are no reports on the susceptibility to infection of glandular cells, although hormonal disarranges have been found. Our results indicate a gradient of molecular interactions, and therefore a possible explanation of susceptibility to infection. An hypothesis of a gradient of susceptibility to infection is supported by the availability of entry proteins, the concordance of connectivity, and previous autopsy and clinical reports.

Several of the cells herein identified having a high potential connectivity with viral proteins were expected given the clinical and pathological observations. For example, part of the oral system^25^, digestive tract, or pneumocytes. Others like cerebral cortex, skin, appendix (glandular cells), endometrium, and tonsils, were not expected and should be considered for further investigation. Furthermore, the amplitude in tropism could contribute to the diversity of symptoms, severity^26^ or long^27,28^ COVID. It is also possible that in some cases, by having a mild capacity to infect certain cells (due to a low PPI connectivity), no symptoms occur; however, due to overactivation of the immune system and cell lysis, several autoantigens may be exposed and facilitate the progress from a mild or moderate disease to a long COVID-19^29^.

A relevant finding that merits further investigation in both the acute disease and the long COVID syndrome is the possible deregulation of ribosome biogenesis, due to its critical role in cell function and dysfunction in diseases such as cancer^30^. In addition to intracellular molecular alterations, such as ribosome biogenesis, already reported in SARS-CoV-1 by PPI^31^, we show a set of coagulation cascade proteins interacting directly and indirectly with SARS-CoV-2 proteins, which could promote their dysregulation. These findings, together with pro-thrombotic autoantibodies^26^ and other potential causes^32^, may account for explaining a coagulation-promoting environment affecting different organs^33^.

A relevant result for SARS-CoV-1 was that lung macrophages could be identified as cells in which the molecular environment may not be significantly altered, and therefore, cell sequestration would be inefficient. This agrees with previous research^34,35^, where it was identified that the viral particle could enter that cell but not replicate, which may also be occurring with several SARS-CoV-2 infected cells, such as tonsil’s squamous epithelial cells.

For SARS-CoV-1 we identified that pneumocytes were almost isolated from the rest of the cells (Fig. 7b). Only a few more cells have the same connectivity: endometrial glandular cells, esophagus squamous epithelial cells, and tonsil’s non-germinal center cells. In contrast, for SARS-CoV-2 there are a significant number of cells closely connected to pneumocytes (Fig. 7a), which may reflect the multi-tropism that it has shown. Another point of contrast is the connectivity shown by both virus types, higher in SARS-CoV-1; although this may be due to properties of the networks, it may also reflect a greater capacity of SARS-CoV-1 to take control of the target cells, which would possibly account for its greater lethality.

This approach sheds light on several SARS-CoV-2 target cells and could be very useful in studying the new variants of interest. For example, some mutations in the spike protein (S) confer the virus a better binding to ACE2^36^, without modifying its tropism. However, in the case of Omicron, its tropism for pneumocytes seems to have been altered. Further research is warranted to assess how the PPI have been modified.

In our work we did not consider using directed networks, given their still limited coverage. Although it would give us valuable information about “indispensable” nodes^37^ directly associated with the virus, the loss of information would limit the results, especially in limited virus-host PPIs. With our strategy we obtained the highly connected nodes (hubs) (supplementary tables S3 and S4).

In conclusion, we identified the connectivity gradient of a set of 55 cell types whose molecular environment may be altered by SARS-CoV-2. This approach allowed us to detect their potential susceptibilty to be controlled by the virus. As expected, pneumocytes were amongst them and, noteworthy, also glandular cells. We also identified several proteins of the coagulation cascade that may be directly and indirectly affected by viral proteins, which could help explain the coagulation disorders associated with this infection. Other possible targets of infection we identified were cells from the small intestine, colon, stomach, and pancreas; heart, kidney, salivary glands, appendix, and tonsils; testicles, prostate, and endometrium among others, not necessarily expected. We have also identified a profile of molecular alterations in the form of a gradient of infection. This model is still perfectible, but it represents an approach to determine the cell tropism and cell-affecting mechanisms of SARS-CoV-2 and other viruses, such as SARS-CoV-1. We expect this model to contribute to the fight against current and future pandemics by identifying both virus and host proteins essential for infection, regardless limited PPI virus-host information.

## Methods

### PPIs

The PPIs between SARS-CoV-2 and human proteins were collected from IMEX^38^, and those of SARS-CoV-1, from Biogrid^39^. Because the present strategy addresses both limited and numerous cases of virus-host PPI, the confidence value was not considered. On the other hand, the human interactome was generated from the compilation of information from several primary databases^39–45^. Only PPIs that were reported in at least two studies were considered.

### Proteomes and transcriptomes

From the Normal tissue and RNA Consensus Tissue Gene databases, deposited in the Protein Atlas (http://www.proteinatlas.org)^46^, proteomes corresponding to each cell of each tissue and transcriptomes of each tissue were generated. Only those tissue genes/proteins with transcriptomic and proteomic evidence were considered (see Fig. 1). Proteins with an uncertain reliability score and genes with normalized expression (NX) values less than one were eliminated. For normal tissue data (cells), values of 1, 2, 3 and 4 were assigned (provided there was a value ≥ 1 in RNA consensus tissue gene data), for Not detected, Low, Medium, and High, respectively for expression value (level). For RNA consensus tissue gene data, the assigned NX values were used. The expression value and NX values of each cell were plotted by unsupervised hierarchical clustering in R^47^ using the gplots library^48^.

### Entrance tropism

To identify which cells and tissues have the components to allow viral entry, we assessed the presence of NRP1^10^, ACE2^11–18^, BSG^11^, TMPRSS2^11–14^, CTSL^11,13^, CTSB^11,13^ and FURIN^11^ proteins, or evidence of their gene expression. Those with a combination of protein S-binding proteins (ACE2, NRP1 or BSG) and proteases necessary for protein S cleavage and membrane fusion (TMPRSS2, CTSL, CTSB or FURIN) were selected. We considered only those tissues with information at both levels (proteome and transcriptome).

### Tropism at PPI level

At this point, only the proteome (cell) data were processed. The interactomes (Virus-Human (V-H) and Human–Human (H–H)) and proteomes of each cell were used. The interactomes of each cell of each tissue were generated considering the interactions of SARS-CoV-2 with human proteins (V-H), a second level of interaction (H–H), and the interactions between them. The networks were visualized with Cytoscape^49^, associating the Expression value and the level of interaction associated with the connectivity of each protein. Enrichment analysis was generated with Enrichr^50^ to identify the alterations of each cell, using the enrichR library of R to automate the procedures. Heatmaps were performed with gplots.

Finally, centroids of each cell were identified by k-means, using the PPIs and the Proteome Expression value of each cell. Next, cell clusters susceptible to SARS-CoV-2 infection were identified according to its connectivity and autopsy reports.

All the methods were performed in accordance with relevant guidelines and regulations.

### Limitations

The choice not to consider a confidence value of virus-host PPIs might generate some uncertainty, but some PPIs with low confidence values may actually represent host-protein interactions. We are aware that a stricter choice of PPIs could modify our conclusions; however, the objective of our strategy was to present a tool to obtain quick answers in an initial context of little information.

Several cells that have been reported to have clinical significance were not considered, due to a lack of information about them at the transcriptome or proteome level. The lack of information on certain cell types and of availability of proteome or transcriptome information meant that not all expected results were obtained, as well as some other hypothetical ones. Cells with a small proteome, such as ovarian (follicular cells) or placental (decidual cells) were also discarded. Whether this is due to a truly small proteome or a technical inability to identify various proteins in these cells remains to be determined.

## Supplementary Information

Supplementary Information 1.

Supplementary Information 2.

## Data Availability

The datasets analyzed during the current study are available in the IMEx repository, https://www.ebi.ac.uk/intact/imex/main.xhtml;jsessionid=49BA95180925C722BEE62ADE77D78873?conversationContext=1; DIP repository, https://dip.doe-mbi.ucla.edu/dip/File.cgi?FN=2016/imex/dip_imex20161230CT2016.xml; IntAct (and Mint) repository, https://www.ebi.ac.uk/intact/search?query=9606&interactorSpeciesFilter=Homo%20sapiens; BioGRID repository, https://downloads.thebiogrid.org/Download/BioGRID/Release-Archive/BIOGRID-4.4.209/BIOGRID-ALL-4.4.209.mitab.zip; HPRD repository, http://hprd.org/download; MatrixDB repository, matrixdb.univ-lyon1.fr/download/matrixdb_FULL.tab.gz; Reactome repository, https://reactome.org/download/current/interactors/reactome.homo_sapiens.interactions.psi-mitab.txt; and HPA repository, https://www.proteinatlas.org/download/normal_tissue.tsv.zip.
