# Supplementary material for: An approach to cellular tropism of SARS-CoV-2 through protein–protein interaction and enrichment analysis
Source: Sci Rep. 2022 Jun 7;12:9399. doi: 10.1038/s41598-022-13625-z (PMC9172986; doi:10.1038/s41598-022-13625-z)
Supplement: Supplementary file 1 — Supplementary Information 1. [file 41598_2022_13625_MOESM1_ESM.pdf]

An approach to cellular tropism of SARS-CoV-2 through protein-protein interaction and enrichment analysis

Daniel Ortega-Bernal, Selene Zarate, Maria de los Ángeles Martinez-Cardenas and Rafael Bojalil*

*Correspondence: rbojalil@correo.xoc.uam.mx

Supplementary Information

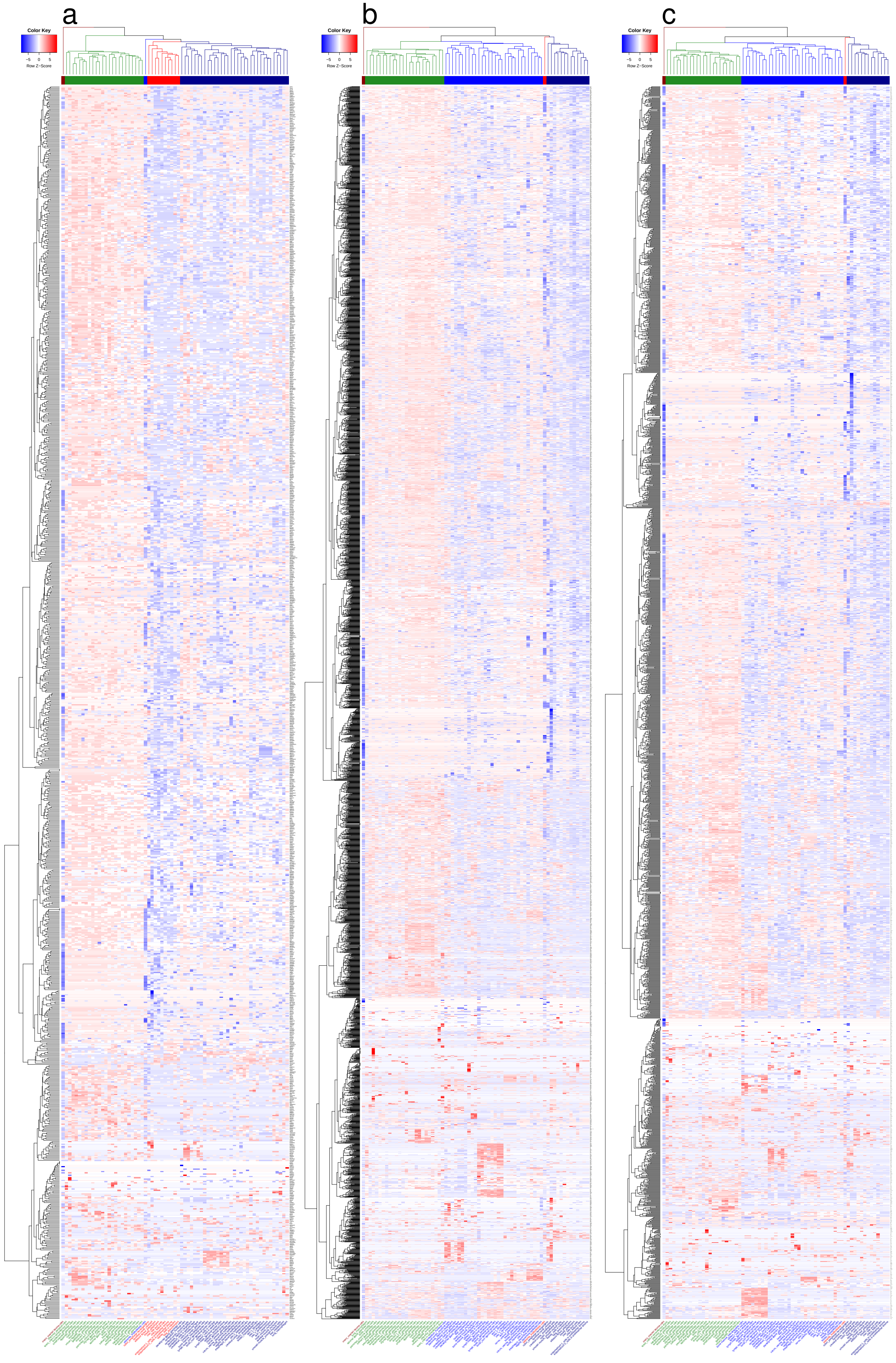
a
b
c
Color Key
−5 0 5
Row Z−Score

Figure S1. Heatmap proteomes. (a) Human proteins that interact with SARS-CoV-2 proteins (V-H), 990. (b) Second level of interaction (H-H), 3,286. (c) V-H and H-H proteins (1,852) in SARS-CoV-1. In the three figures green branch is mainly represented by glandular cells, which show overexpression with respect to the rest of the cells.

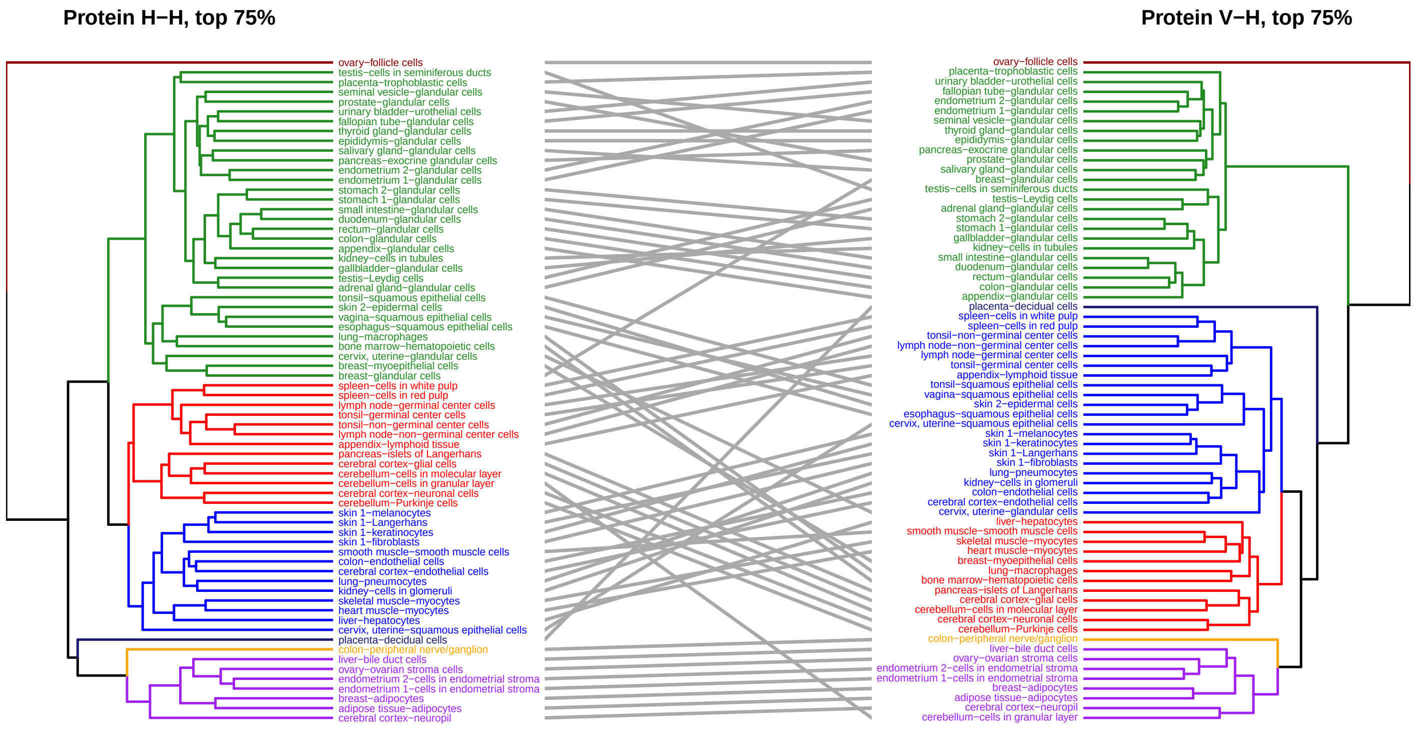

Figure S2. Dendrograms comparison of both groups of proteins (V-H and H-H) in SARS-CoV-2. Selecting the 75% of the most discriminating proteins. Green branch corresponds to green branch in Figure S1, mainly glandular cells and with higher expression than the other cells.

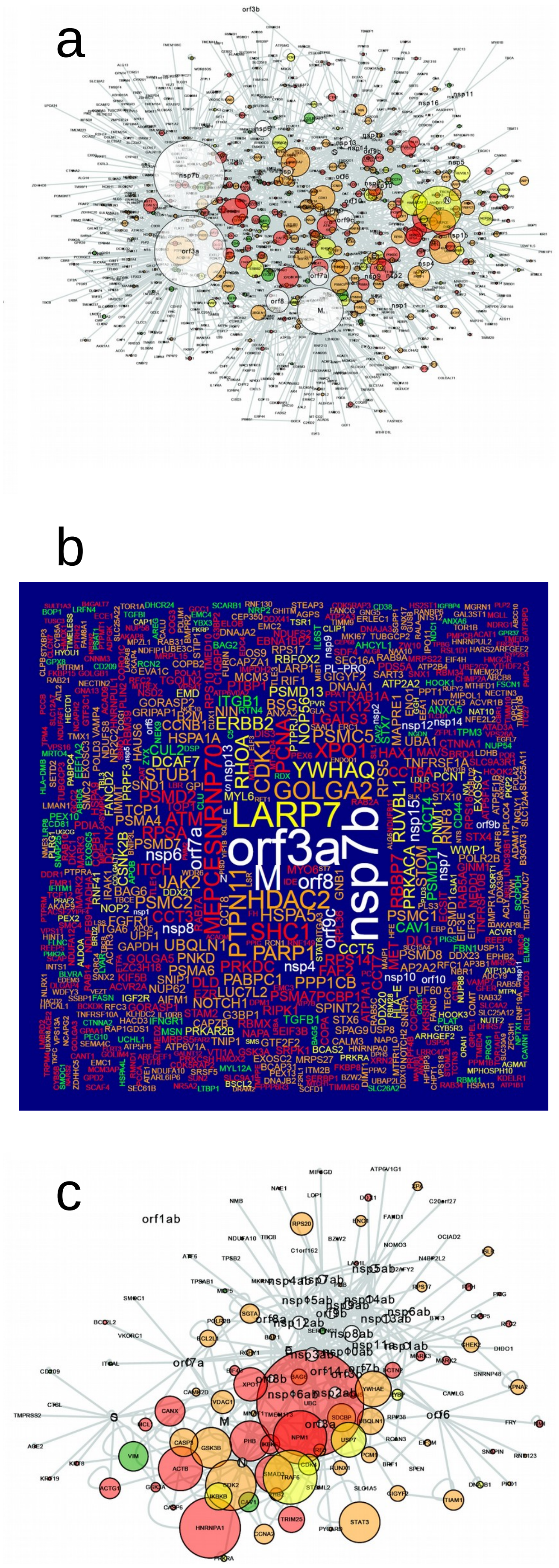
a
b
c

Figure S3. V-H networks and wordcloud (Complement to Fig. 3). (a) and (c) small intestine (glandular cells) networks in SARS-CoV-2 and SARS-CoV-1 respectively. (b) wordclouds show main proteins present in (a). The size of the circles or words indicates connectivity and the color to the Expression value, green: only present at transcript level, yellow: low amount of protein reported, orange: intermediate amount of protein, red: high amount of protein, and in white the viral proteins.

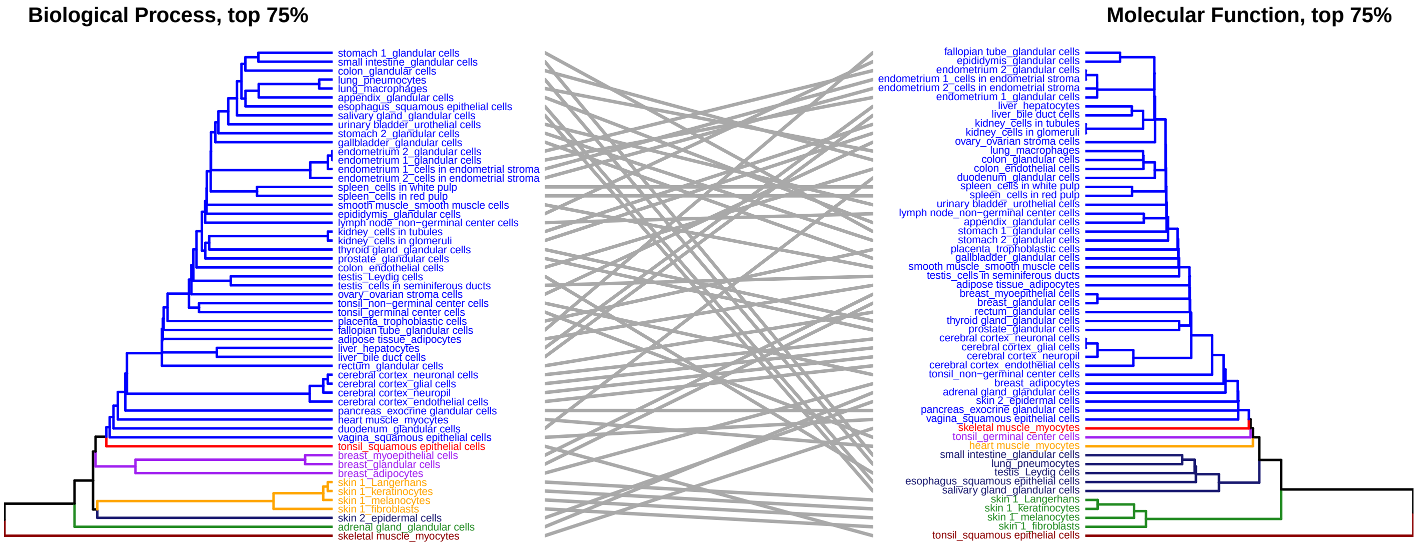

Figure S4. Dendrogram comparison of 75% most discriminating molecular functions and biological processes in SARS-CoV-2. Previously, 14 cell types were removed. In this comparison it is identified that Skin 1 cells show the least alteration, yellow branches in biological processes and green in molecular functions: as well as squamous epithelial cells (tonsil).

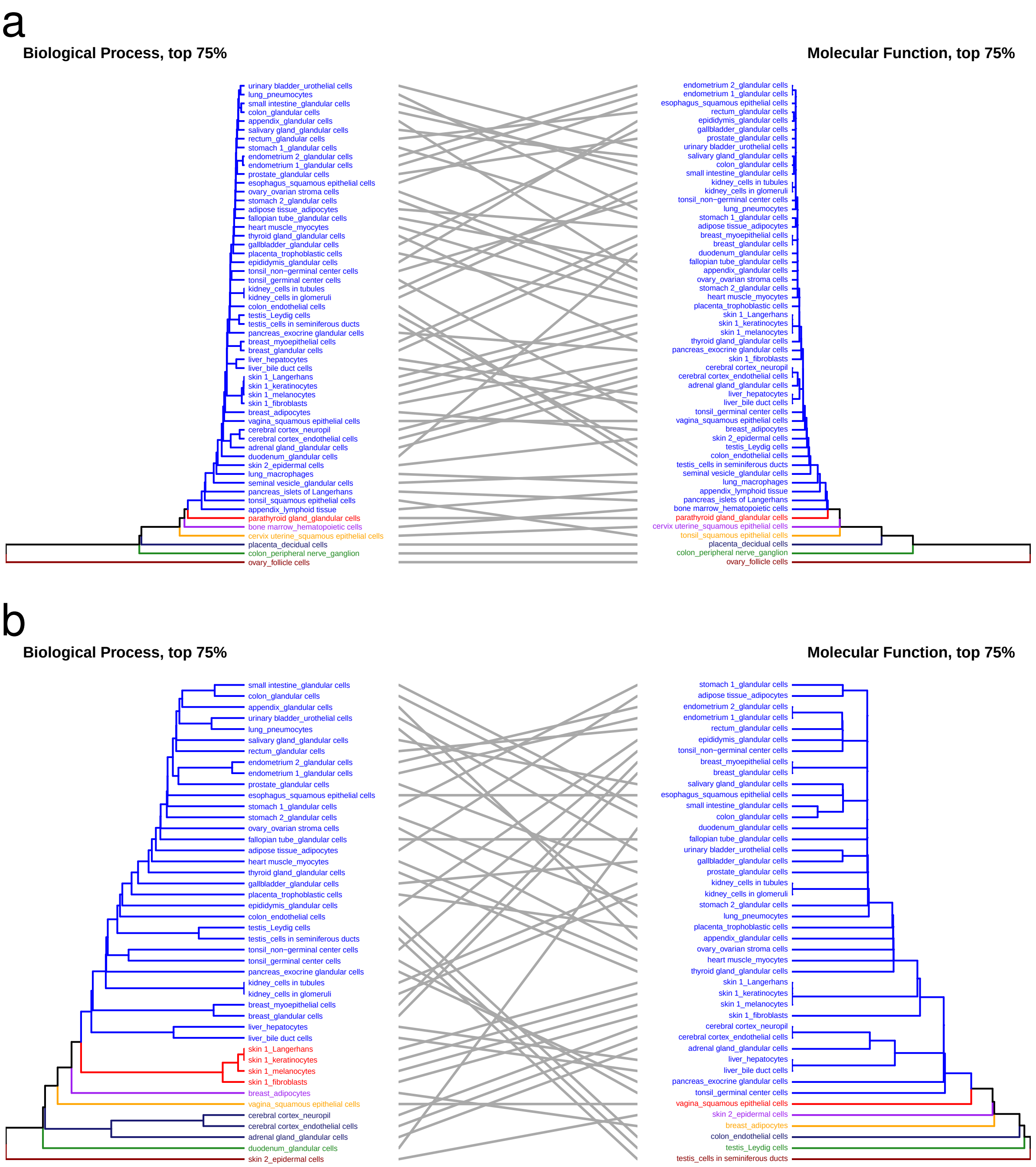

Figure S5. Comparison of 75% of the most discriminating molecular functions and biological processes between SARS-CoV-1 cells. (a) After removing cells only with a report of mRNA for input proteins; are identifiable, in the bottom branches, cells that due to the effect of proteome size do not obtain similar results to cells with average size proteome, as well as macrophages (lung) and squamous epithelial cells (tonsil). (b) After removing cells that have a proteome size effect; some awareness is observed in bottom branches cells, but no cells were removed at this level.

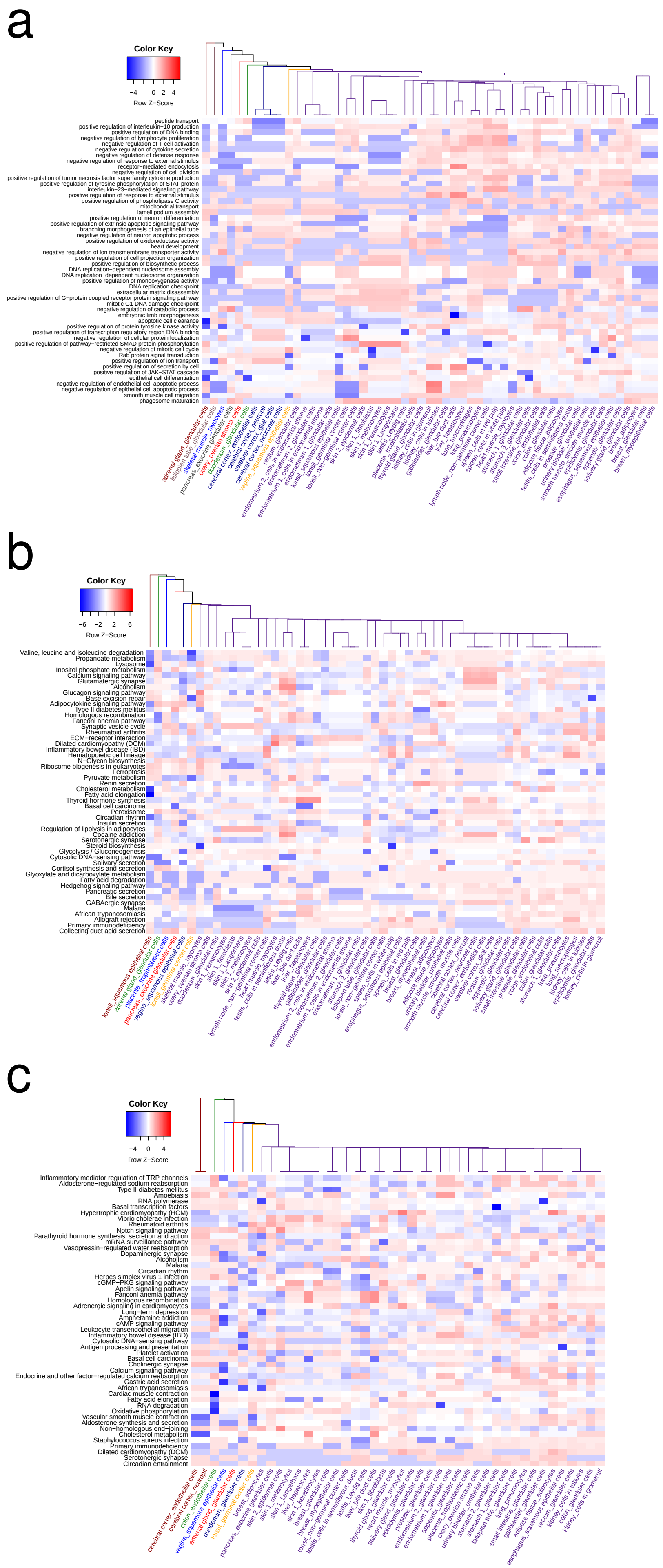
a
Color Key
−4 0 2 4
Row Z−Score
peptide transport
positive regulation of interleukin−10 production
positive regulation of DNA binding
negative regulation of lymphocyte proliferation
negative regulation of T cell activation
negative regulation of cytokine secretion
negative regulation of defense response
negative regulation of response to external stimulus
receptor−mediated endocytosis
negative regulation of cell division
positive regulation of tumor necrosis factor superfamily cytokine production
positive regulation of tyrosine phosphorylation of STAT protein
interleukin−23−mediated signaling pathway
positive regulation of response to external stimulus
positive regulation of phospholipase C activity
mitochondrial transport
lamellipodium assembly
positive regulation of neuron differentiation
positive regulation of extrinsic apoptotic signaling pathway
branching morphogenesis of an epithelial tube
negative regulation of neuron apoptotic process
positive regulation of oxidoreductase activity
heart development
negative regulation of ion transmembrane transporter activity
positive regulation of cell projection organization
positive regulation of biosynthetic process
DNA replication−dependent nucleosome assembly
DNA replication−dependent nucleosome organization
positive regulation of monooxygenase activity
extracellular matrix disassembly
positive regulation of G−protein coupled receptor protein signaling pathway
DNA replication checkpoint
mitotic G1 DNA damage checkpoint
negative regulation of catabolic process
embryonic limb morphogenesis
apoptotic cell clearance
positive regulation of protein tyrosine kinase activity
positive regulation of transcription regulatory region DNA binding
negative regulation of cellular protein localization
positive regulation of pathway−restricted SMAD protein phosphorylation
negative regulation of mitotic cell cycle
Rab protein signal transduction
positive regulation of ion transport
positive regulation of secretion by cell
positive regulation of JAK−STAT cascade
epithelial cell differentiation
negative regulation of endothelial cell apoptotic process
negative regulation of epithelial cell apoptotic process
smooth muscle cell migration
phagosome maturation
b
Color Key
−6 −2 2 6
Row Z−Score
Valine, leucine and isoleucine degradation
Propanoate metabolism
Lysosome
Inositol phosphate metabolism
Calcium signaling pathway
Glutamatergic synapse
Alcoholism
Glucagon signaling pathway
Base excision repair
Adipocytokine signaling pathway
Type II diabetes mellitus
Homologous recombination
Fanconi anemia pathway
Synaptic vesicle cycle
Rheumatoid arthritis
ECM−receptor interaction
Dilated cardiomyopathy (DCM)
Inflammatory bowel disease (IBD)
Hematopoietic cell lineage
N−Glycan biosynthesis
Ribosome biogenesis in eukaryotes
Ferroptosis
Pyruvate metabolism
Renin secretion
Cholesterol metabolism
Fatty acid elongation
Thyroid hormone synthesis
Basal cell carcinoma
Peroxisome
Circadian rhythm
Insulin secretion
Regulation of lipolysis in adipocytes
Cocaine addiction
Serotonergic synapse
Steroid biosynthesis
Glycolysis / Gluconeogenesis
Cytosolic DNA−sensing pathway
Salivary secretion
Cortisol synthesis and secretion
Glyoxylate and dicarboxylate metabolism
Fatty acid degradation
Hedgehog signaling pathway
Pancreatic secretion
Bile secretion
GABAergic synapse
Malaria
African trypanosomiasis
Allograft rejection
Primary immunodeficiency
Collecting duct acid secretion
c
Color Key
−4 0 4
Row Z−Score
Inflammatory mediator regulation of TRP channels
Aldosterone−regulated sodium reabsorption
Type II diabetes mellitus
Amoebiasis
RNA polymerase
Basal transcription factors
Hypertrophic cardiomyopathy (HCM)
Vibrio cholerae infection
Rheumatoid arthritis
Notch signaling pathway
Parathyroid hormone synthesis, secretion and action
mRNA surveillance pathway
Vasopressin−regulated water reabsorption
Dopaminergic synapse
Alcoholism
Malaria
Circadian rhythm
Herpes simplex virus 1 infection
cGMP−PKG signaling pathway
Apelin signaling pathway
Fanconi anemia pathway
Homologous recombination
Adrenergic signaling in cardiomyocytes
Long−term depression
Amphetamine addiction
cAMP signaling pathway
Leukocyte transendothelial migration
Inflammatory bowel disease (IBD)
Cytosolic DNA−sensing pathway
Antigen processing and presentation
Platelet activation
Basal cell carcinoma
Cholinergic synapse
Calcium signaling pathway
Endocrine and other factor−regulated calcium reabsorption
Gastric acid secretion
African trypanosomiasis
Cardiac muscle contraction
Fatty acid elongation
RNA degradation
Oxidative phosphorylation
Vascular smooth muscle contraction
Aldosterone synthesis and secretion
Non−homologous end−joining
Cholesterol metabolism
Staphylococcus aureus infection
Primary immunodeficiency
Dilated cardiomyopathy (DCM)
Serotonergic synapse
Circadian entrainment

Figure S6. Enrichment results, reduction. (a) Heatmap of the 50 BP most discriminant in SARS-CoV-2. (b) Heatmap of the 50 most discriminant signaling pathways in SARS-CoV-2. (c) Heatmap of the 50 most discriminant signaling pathways in SARS-CoV-1. After reduction to 55 and 45 cells of SARS-CoV-2 and SARS-CoV-1, the gradient is still preserved, identifying pneumocytes as one of the most affected cells in both viruses.

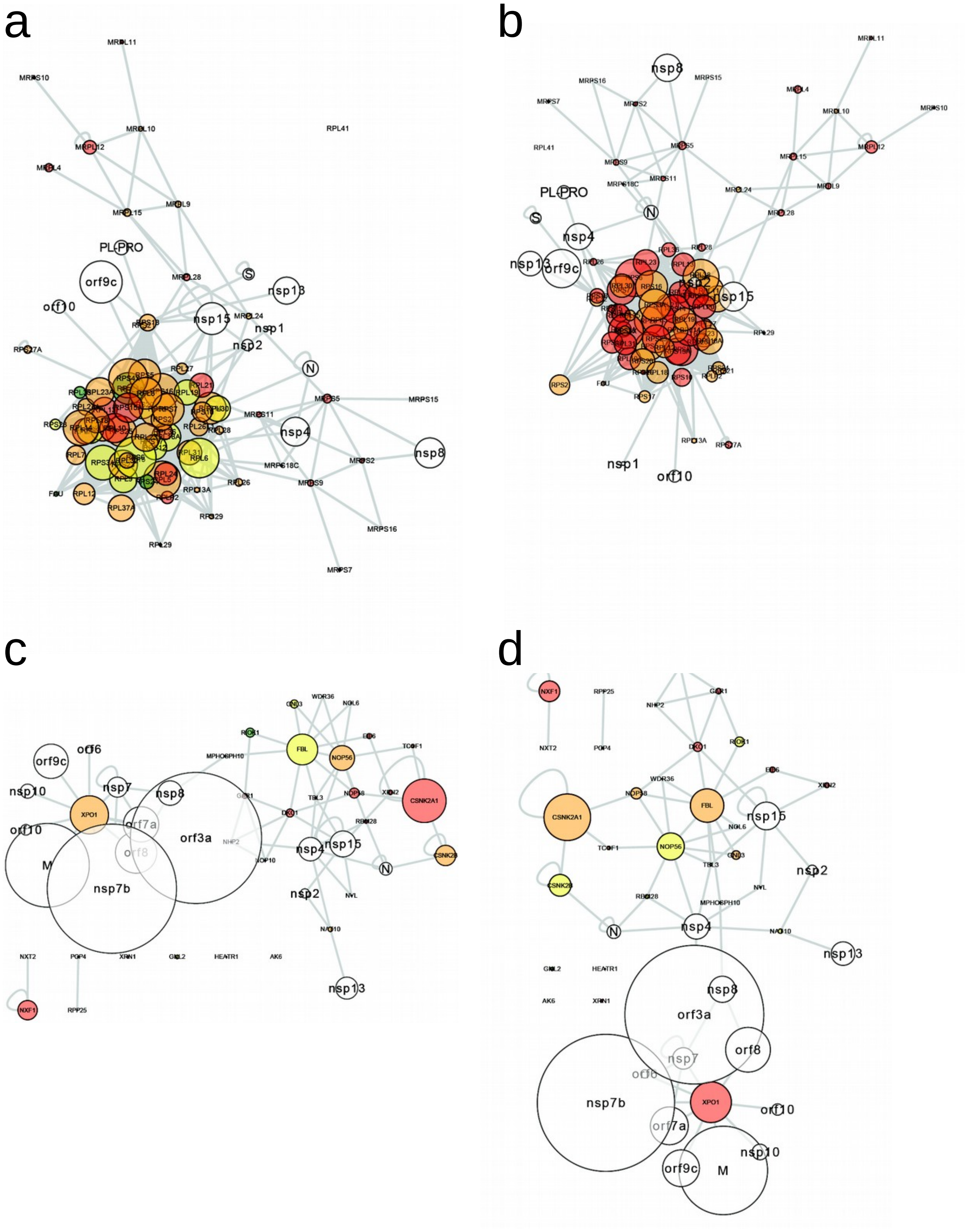

Figure S7. (a) and (b) ribosome pathway networks; (c) and (d) networks of the ribosome synthesis pathway. Kidney (cells in tubules), small intestine (glandular cells), respectively. Complement to Fig. 5.

References (Supplementary tables S1 and S2)

1. Bian, X.-W. COVID-19 Pathology Team. Autopsy of COVID-19 patients in China. Natl. Sci. Rev. 7, 1414–1418 (2020).

2. Best Rocha, A. et al. Detection of SARS-CoV-2 in formalin-fixed paraffin-embedded tissue sections using commercially available reagents. Lab. Investig. J. Tech. Methods Pathol. 100, 1485–1489 (2020).

3. Skok, K., Stelzl, E., Trauner, M., Kessler, H. H. Lax, S. F. Post-mortem viral dynamics and tropism in COVID-19 patients in correlation with organ damage. Virchows Arch. 478, 343–353 (2021).

4. Deinhardt-Emmer, S. et al. Early postmortem mapping of SARS-CoV-2 RNA in patients with COVID-19 and the correlation with tissue damage. eLife 10, e60361 (2021).

5. Remmelink, M. et al. Unspecific post-mortem findings despite multiorgan viral spread in COVID-19 patients. Crit. Care Lond. Engl. 24, 495 (2020).

6. Hanley, B. et al. Histopathological findings and viral tropism in UK patients with severe fatal COVID-19: a post-mortem study. Lancet Microbe 1, e245–e253 (2020).

7. Liu, J. et al. SARS-CoV-2 cell tropism and multiorgan infection. Cell Discov. 7, 17 (2021).

8. Huang, N. et al. Integrated Single-Cell Atlases Reveal an Oral SARS-CoV-2 Infection and Transmission Axis. MedRxiv Prepr. Serv. Health Sci. 2020.10.26.20219089 (2020) doi:10.1101/2020.10.26.20219089.

9. Jamiolkowski, D. et al. SARS-CoV-2 PCR testing of skin for COVID-19 diagnostics: a case report. Lancet Lond. Engl. 396, 598–599 (2020).

10. Bradley, B. T. et al. Histopathology and ultrastructural findings of fatal COVID-19 infections in Washington State: a case series. Lancet Lond. Engl. 396, 320–332 (2020).

11. Gomes, I. C. et al. SARS-CoV-2 Infection in the Central Nervous System of a 1-Year-Old Infant Submitted to Complete Autopsy. (2020) doi:10.20944/preprints202009.0297.v1.

12. Schurink, B. et al. Viral presence and immunopathology in patients with lethal COVID-19: a prospective autopsy cohort study. Lancet Microbe 1, e290–e299 (2020).

13. Lin, L. et al. Gastrointestinal symptoms of 95 cases with SARS-CoV-2 infection. Gut 69, 997–1001 (2020).

14. Wichmann, D. et al. Autopsy Findings and Venous Thromboembolism in Patients With COVID-19: A Prospective Cohort Study. Ann. Intern. Med. 173, 268–277 (2020).

15. Yang, M. et al. Pathological Findings in the Testes of COVID-19 Patients: Clinical Implications. Eur. Urol. Focus 6, 1124–1129 (2020).

16. Achua, J. K. et al. Histopathology and Ultrastructural Findings of Fatal COVID-19 Infections on Testis. World J. Mens Health 39, 65–74 (2021).

17. Ma, X. et al. Pathological and molecular examinations of postmortem testis biopsies reveal SARS-CoV-2 infection in the testis and spermatogenesis damage in COVID-19 patients. Cell. Mol. Immunol. 18, 487–489 (2021).

18. Wang, Y. et al. SARS-CoV-2 infection of the liver directly contributes to hepatic impairment in patients with COVID-19. J. Hepatol. 73, 807–816 (2020).

19. Patanè, L. et al. Vertical transmission of coronavirus disease 2019: severe acute respiratory syndrome coronavirus 2 RNA on the fetal side of the placenta in pregnancies with coronavirus disease 2019-positive mothers and neonates at birth. Am. J. Obstet. Gynecol. MFM 2, 100145 (2020).

20. Hosier, H. et al. SARS-CoV-2 infection of the placenta. J. Clin. Invest. 130, 4947–4953 (2020).

21. Carsana, L. et al. Pulmonary post-mortem findings in a series of COVID-19 cases from northern Italy: a two-centre descriptive study. Lancet Infect. Dis. 20, 1135–1140 (2020).

22. Ramos da Silva, S. et al. Broad Severe Acute Respiratory Syndrome Coronavirus 2 Cell Tropism and Immunopathology in Lung Tissues From Fatal Coronavirus Disease 2019. J. Infect. Dis. 223, 1842–1854 (2021).

23. Puelles, V. G. et al. Multiorgan and Renal Tropism of SARS-CoV-2. N. Engl. J. Med. 383, 590–592 (2020).

24. Bhatnagar, J. et al. Evidence of Severe Acute Respiratory Syndrome Coronavirus 2 Replication and Tropism in the Lungs, Airways, and Vascular Endothelium of Patients With Fatal Coronavirus Disease 2019: An Autopsy Case Series. J. Infect. Dis. 223, 752–764 (2021).

25. Braun, F. et al. SARS-CoV-2 renal tropism associates with acute kidney injury. Lancet Lond. Engl. 396, 597–598 (2020).

26. Su, H. et al. Renal histopathological analysis of 26 postmortem findings of patients with COVID-19 in China. Kidney Int. 98, 219–227 (2020).

27. Farcas, G. A. et al. Fatal severe acute respiratory syndrome is associated with multiorgan involvement by coronavirus. J. Infect. Dis. 191, 193–197 (2005).

28. Ding, Y. et al. Organ distribution of severe acute respiratory syndrome (SARS) associated coronavirus (SARS-CoV) in SARS patients: implications for pathogenesis and virus transmission pathways. J. Pathol. 203, 622–630 (2004).

29. Gu, J. et al. Multiple organ infection and the pathogenesis of SARS. J. Exp. Med. 202, 415–424 (2005).

30. Nicholls, J. M. et al. Time course and cellular localization of SARS-CoV nucleoprotein and RNA in lungs from fatal cases of SARS. PLoS Med. 3, e27 (2006).

31. Leung, W. K. et al. Enteric involvement of severe acute respiratory syndrome-associated coronavirus infection. Gastroenterology 125, 1011–1017 (2003).

32. To, K. F. et al. Tissue and cellular tropism of the coronavirus associated with severe acute respiratory syndrome: an in-situ hybridization study of fatal cases. J. Pathol. 202, 157–163 (2004).

33. Nicholls, J. M. et al. Lung pathology of fatal severe acute respiratory syndrome. Lancet Lond. Engl. 361, 1773–1778 (2003).

34. Franks, T. J. et al. Lung pathology of severe acute respiratory syndrome (SARS): a study of 8 autopsy cases from Singapore. Hum. Pathol. 34, 743–748 (2003).

35. Lang, Z.-W. et al. A clinicopathological study of three cases of severe acute respiratory syndrome (SARS). Pathology (Phila.) 35, 526–531 (2003).

36. Chow, K.-C., Hsiao, C.-H., Lin, T.-Y., Chen, C.-L. Chiou, S.-H. Detection of Severe Acute Respiratory Syndrome–Associated Coronavirus in Pneumocytes of the Lung. Am. J. Clin. Pathol. 121, 574–580 (2004).
